# Resveratrol Attenuates Copper-Induced Senescence by Improving Cellular Proteostasis

**DOI:** 10.1155/2017/3793817

**Published:** 2017-02-09

**Authors:** Liliana Matos, Alexandra Monteiro Gouveia, Henrique Almeida

**Affiliations:** ^1^Departamento de Biologia Experimental, Faculdade de Medicina, IBMC, Instituto de Biologia Molecular e Celular and I3S, Instituto de Investigação e Inovação em Saúde, Universidade do Porto, Alameda Prof. Hernâni Monteiro, 4200-319 Porto, Portugal; ^2^Faculdade de Ciências da Nutrição e Alimentação, Universidade do Porto, Rua Dr. Roberto Frias, 4200-465 Porto, Portugal

## Abstract

Copper sulfate-induced premature senescence (CuSO_4_-SIPS) consistently mimetized molecular mechanisms of replicative senescence, particularly at the endoplasmic reticulum proteostasis level. In fact, disruption of protein homeostasis has been associated to age-related cell/tissue dysfunction and human disorders susceptibility. Resveratrol is a polyphenolic compound with proved antiaging properties under particular conditions. In this setting, we aimed to evaluate resveratrol ability to attenuate cellular senescence induction and to unravel related molecular mechanisms. Using CuSO_4_-SIPS WI-38 fibroblasts, resveratrol is shown to attenuate typical senescence alterations on cell morphology, senescence-associated beta-galactosidase activity, and cell proliferation. The mechanisms implicated in this antisenescence effect seem to be independent of senescence-associated genes and proteins regulation but are reliant on cellular proteostasis improvement. In fact, resveratrol supplementation restores copper-induced increased protein content, attenuates BiP level, and reduces carbonylated and polyubiquitinated proteins by autophagy induction. Our data provide compelling evidence for the beneficial effects of resveratrol by mitigating CuSO_4_-SIPS stressful consequences by the modulation of protein quality control systems. These findings highlight the importance of a balanced cellular proteostasis and add further knowledge on molecular mechanisms mediating resveratrol antisenescence effects. Moreover, they contribute to identifying specific molecular targets whose modulation will prevent age-associated cell dysfunction and improve human healthspan.

## 1. Introduction

Normal somatic dividing cells have been proved to be valuable in vitro models to study cellular senescence and unravel molecular mechanisms and pathways implicated in the human aging process. The well-known model of replicative senescence (RS) is achieved when human diploid fibroblasts (HDFs) spontaneously stop dividing after an initial active period of population doublings and become unresponsive to mitogenic stimuli [1]. Besides the irreversible cell cycle arrest, RS fibroblasts exhibit other typical, morphological, and molecular features, such as increased cellular volume, higher senescence-associated beta-galactosidase (SA beta-gal) activity, and increased expression of senescence-associated genes and proteins [2, 3]. A similar senescent phenotype, termed stress-induced premature senescence (SIPS), can be attained by the exposure of HDFs to subcytotoxic doses of oxidative stress inducers such as hydrogen peroxide (H_2_O_2_-SIPS) [4], tert-butyl hydroperoxide, ultraviolet B radiation [3], or copper sulfate (CuSO_4_-SIPS) [5]. Recently, the latter was shown to mimic better the RS model compared to the most frequently used H_2_O_2_-SIPS model [6].

Resveratrol is a natural polyphenolic compound that was shown to increase maximum lifespan of several organisms, such as* Saccharomyces cerevisiae *[7],* Caenorhabditis elegans *[8],* Drosophila melanogaster* [9], and the short-lived fish* Nothobranchius furzeri *[10]. Yet, resveratrol failed to extend longevity in rodent mammals, even though it improved their healthspan, thus evidencing a protective role against age-related deterioration [11].

At the cellular level, resveratrol has also been shown to attenuate senescence features in either RS [12] or H_2_O_2_-SIPS [13, 14] cellular models. These antiaging effects have long been associated with resveratrol ability to activate sirtuin 1 deacetylase, Sirt1 [for a review see [15]]. Actually, it was demonstrated that Sirt1 overexpression attenuates senescence and extends replicative lifespan of several cultured cell types [16–18], while its inhibition results in increased cellular senescence [16]. Also, Sirt1 was shown to be downregulated with aging [19] and in cellular senescence models [20, 21] further favoring its preventive role of senescence features. Besides resveratrol ability to modulate signal transduction pathways through the activation of Sirt1 [14, 22], several other biological events were assigned as responsible for its positive effects, including its ability to increase stress resistance [12], to induce telomerase activity [23], to decrease the secretion of senescence-associated proinflammatory proteins [24], and to inhibit the mechanistic target of rapamycin, mTOR [13]. Resveratrol was also found to modulate protein quality control cellular responses, as it was shown to regulate the expression of the heat shock molecular chaperones [25] and to promote cellular protein degradation mechanisms, namely, ubiquitin-proteasome system (UPS) [26, 27] and lysosomal autophagy [28, 29]. Moreover, resveratrol was able to increase* C. elegans* lifespan through the upregulation of* abu11* (activated in blocked unfolded protein response-11), which encodes a protein involved in the endoplasmic reticulum (ER) unfolded protein response (UPR) that protects the organism from damage by improperly folded proteins [8].

In the present study we aimed to evaluate the ability of resveratrol to attenuate the establishment of cellular senescence upon CuSO_4_ induction, unravelling the molecular mechanisms that might be involved. It was found that resveratrol supplementation was able to reduce the appearance of some senescence-associated features by the improvement of cellular proteostasis probably by protecting proteins from oxidative damage and preventing their accumulation by the induction of protein degradation mechanisms.

## 2. Material and Methods

### 2.1. Cell Culture

WI-38 human fetal lung fibroblasts were purchased from The European Collection of Cell Cultures (ECACC) and were cultivated in complete medium composed of Basal Medium Eagle (BME) supplemented with 10% fetal bovine serum, at 37°C in a 5% CO_2_ humidified atmosphere. WI-38 cells are considered to be young below 30 population doublings (PDs) and enter senescence at 45 PDs or above. For the induction of SIPS with copper sulfate (CuSO_4_-SIPS), subconfluent young WI-38 fibroblasts were exposed to 350 *μ*M CuSO_4_ (Na_2_SO_4_ for controls) for 24 h. Then, cells were washed once with phosphate buffered saline (PBS) and replaced with fresh complete medium containing 5 or 10 *μ*M of resveratrol (R5010-Sigma-Aldrich®) for an additional 72 h period. Control cells were submitted to a final concentration of 0.1% DMSO for the same period.

### 2.2. Cell Morphology and SA Beta-Gal Detection

Cell morphology evaluation was performed 72 h after copper removal by optical inspection using an inverted microscope. To assess the presence of senescent cells, SA beta-gal was detected 72 h after copper removal as already described [5]. The percentage of SA beta-gal positive cells in each condition was determined by microscopically counting 400 total cells/well from at least three independent experiments.

### 2.3. Cell Proliferation and Total Protein Content

To assess the effect of the different treatments on cell proliferation and total protein content, cell number determination and SRB assay [30] were performed along time after copper removal. Briefly, 3000 cells/well were seeded in 96-well culture plates, treated for 24 h with CuSO_4_ (or Na_2_SO_4_ for controls), and then analyzed at different time-points (0, 24, 48, and 72 h) while recovering in the presence or absence of resveratrol. For cell number determination, cells were trypsinized and stained with Trypan Blue, and the viable cells were microscopically counted in a Neubauer chamber. The total number of cells per well for each condition at the different time-points was calculated and plotted, assuming that, at *t* = 0, for each condition, cell number equals 1. For the total protein content determination, cells were treated with 10% trichloroacetic acid (TCA), 1 hour at 4°C. The TCA-precipitated proteins fixed at the bottom of the wells were stained for 30 minutes with 0.057% (w/v) SRB in 1% acetic acid solution and then washed four times with 1% acetic acid. Bound dye was solubilized with 10 mM Tris base solution and the absorbance at 510 nm of each well was recorded using a microplate reader (Infinite 200, TECAN).

### 2.4. Real Time PCR

*Gen*e expression experiments were performed 72 h after copper sulfate treatment by real time quantitative PCR (qPCR). Total RNA extracted (PureLink® RNA Mini Kit, Ambion) from cells derived from at least three independent cultures from each condition was converted into cDNA by reverse transcription reaction. Amplification reaction assays contained SYBR Green Mastermix (SYBR® Select Master Mix, Applied Biosystems®), 50 ng cDNA and primers (STAB VIDA, Lda.) at optimal concentrations. The primer sequences were p21, 5′-CTGGAGACTCTCAGGGTCGAA-3′ and 5′-CCAGGACTGCAGGCTTCCT-3′; ApoJ, 5′-GGATGAAGGACCAGTGTGACAAG-3′ and 5′-CAGCGACCTGGAGGGATTC-3′; TGF*β*1, 5′-AGGGCTACCATGCCAACTTCT-3′ and 5′-CCGGGTTATGCTGGTTGTACA-3′; and TATA box binding protein (TBP), 5′-TCAAACCCAGAATTGTTCTCCTTAT-3′ and 5′-CCTGAATCCCTTTAGAATAGGGTAGA-3′. The protocol used for qPCR was 95°C (3 min); 40 cycles of 95°C (15 sec); and 60°C (1 min). qPCR was performed in the StepOnePlus™ thermal cycler (Applied Biosystems™). TBP was the selected housekeeping gene when calculating relative transcript levels of the target genes.

### 2.5. Western Blot

Protein levels were assessed 72 h after copper sulfate exposure by western blot analysis. WI-38 cells submitted to the different treatments were washed with PBS and scrapped on ice in a lysis buffer (10 mM Tris, pH 7.4, 100 mM NaCl, 1 mM EDTA, 0.1% Triton X-100) supplemented with protease inhibitors cocktail (Sigma-Aldrich). Upon Bradford assay, 20 *μ*g (or 10 *μ*g for the detection of carbonylated or poly-Ub proteins) of protein from each cell extract was resolved by SDS-PAGE. Proteins were blotted into a nitrocellulose membrane and, after blocking with 5% nonfat dry milk diluted in Tris-buffered saline 0.1% tween 20 (TBST), were probed with specific primary antibodies (anti-HSP90 ab13495 and anti-p62 ab109012, Abcam®; anti-LC3 NB100-2220, Novus Biologicals; anti-ubiquitin PW0930, Enzo® Life Sciences; anti-p21 #2946, anti-phospho-eIF2 #3398, anti-HSP70 #4876, and anti-BiP #3177, Cell Signaling Technology®) overnight at predetermined optimal dilutions. For the specific detection of carbonylated proteins, immediately after protein transfer, the nitrocellulose-bound proteins were treated as described elsewhere [31]. Briefly, the membranes were equilibrated in 20% methanol in TBS, washed for 5 min with 10% trifluoroacetic acid (TFA), derivatized with 5 mM 2,4-dinitrophenylhydrazine (DNPH, Sigma-Aldrich) diluted in 10% TFA for 10 min (protected from light), washed with 10% TFA to remove the excess of DNPH, and finally washed with 50% methanol. Following this procedure, the membranes were blocked with 5% bovine serum albumin in TBST and incubated with primary anti-DNP antibody (D9656, Sigma-Aldrich). From here on, the western blot procedure was similar to all antibodies: after TBST washing, immunoblots were incubated with the appropriate peroxidase-conjugated secondary antibodies for 1 h, detected using ECL western blotting substrate (Pierce™, Thermo Scientific) and visualized in ChemiDocTM XRS (BioRad Laboratories). Results were quantified by densitometry using the Image Lab® software. Protein loading was normalized using Ponceau S protein staining, but similar data were also obtained using tubulin detection (data not shown).

### 2.6. Statistical Analysis

Student's *t*-test was used to compare the means between two different conditions. A *p* value lower than 0.05 was considered statistically significant.

## 3. Results

### 3.1. Sirtuin 1 Expression Is Diminished in CuSO_4_-SIPS

**Figure 1:**
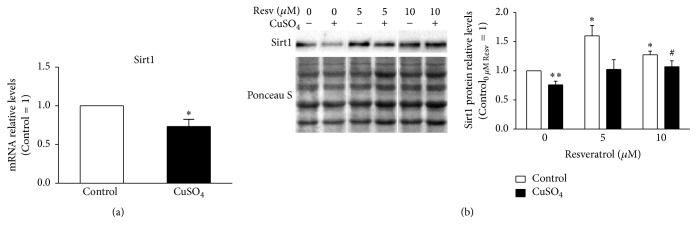
Reduced sirtuin 1 expression in CuSO_4_-SIPS fibroblasts is restored by the addition of resveratrol. (a-b) WI-38 fibroblasts were incubated with 350 *µ*M CuSO_4_ (or Na_2_SO_4_, for controls) for 24 h. Then, media were changed and cells were allowed to recover for an additional 72 h period in the presence of 5 or 10 *µ*M resveratrol (or 0.1% DMSO, for controls). After this recovery period, fibroblasts were processed for different assays. (a) Sirtuin 1 (Sirt1) transcript levels were assessed by qPCR and plotted assuming that mRNA level of controls equals 1. TBP was the selected housekeeping gene. (b) Sirt1 relative protein content was determined by western blot, using Ponceau S staining to normalize protein loading. Depicted blots are representative and densitometric quantification is plotted assuming that control cells in the absence of resveratrol presents a relative protein level of 1. Data represent mean ± SEM of at least three independent experiments. ^*∗*^*p* < 0.05 and ^*∗∗*^*p* < 0.01, when compared to control cells in the absence of resveratrol; ^#^*p* < 0.05, relatively to CuSO_4_-treated cells without resveratrol.

It was already demonstrated that Sirt1 expression decreases with increasing population doublings [20] and also in H_2_O_2_-SIPS cellular models [21]. Here, Sirt1 transcript and protein levels were evaluated by qPCR and western blot, respectively, in CuSO_4_-induced senescent WI-38 fibroblasts. Similarly to other RS and SIPS models, CuSO_4_-SIPS fibroblasts also presented decreased expression of both gene (Figure 1(a)) and protein (Figure 1(b)) Sirt1. Namely, mRNA and protein relative level presented a 27% and 23% reduction, respectively, in copper-treated cells when compared to controls (*p* = 0.04 and *p* = 0.008, resp.). The effect of resveratrol (5 or 10 *μ*M), a Sirt1 activator, was evaluated 72 h after the 24 h incubation of cells with CuSO_4_, which is the usual recovery time that cells need to adapt and develop the senescent phenotype [5]. The addition of 10 *μ*M resveratrol attenuated the copper-induced decrease in Sirt1 protein levels (*p* = 0.047) to values similar to the young control cells. Incubation of non-CuSO_4_ submitted fibroblasts with 5 and 10 *μ*M resveratrol for 72 h increased Sirt1 protein level by 1.6- and 1.3-fold (*p* = 0.008 and *p* = 0.01), respectively, when compared to young control cells (Figure 1(b)).

### 3.2. Resveratrol Attenuates the Appearance of Some Typical Senescence-Associated Alterations

**Figure 2:**
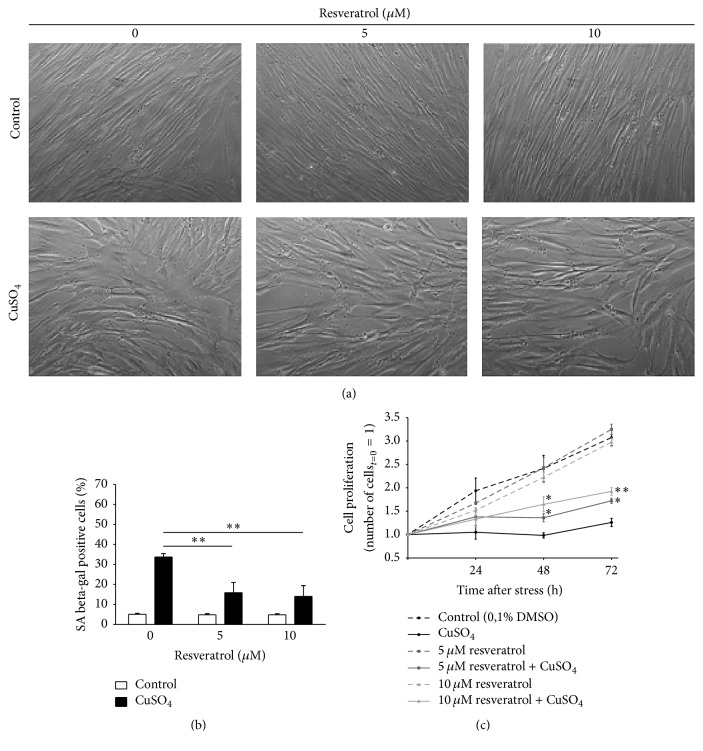
Resveratrol attenuates the appearance of typical senescence-associated features induced by CuSO_4_. (a) Cell morphology was evaluated 72 h after the removal of 350 *µ*M CuSO_4_ (or Na_2_SO_4_, for controls) in fibroblasts that were allowed to recover in the presence or absence of 5 or 10 *µ*M resveratrol. Representative images from the indicated conditions are depicted. (b) Senescence-associated beta-galactosidase (SA beta-gal) activity was detected 72 h after CuSO_4_ removal and the percentage of positive cells was calculated for each condition after counting a minimum of 400 cells/well. (c) Cell proliferation was assessed by counting the viable cells in a Neubauer chamber at different time-points after CuSO_4_ treatment (0, 24, 48, and 72 h). To facilitate direct comparison between the indicated conditions along time, the number of viable cells at day 0 was assumed as 1 for all treatments. Data represent mean ± SEM of at least three independent experiments. ^*∗*^*p* < 0.05 and ^*∗∗*^*p* < 0.01, when compared to CuSO_4_-treated cells without resveratrol at the respective time-point.

Senescent cells usually present typical morphological alterations, increased level of SA beta-gal, and irreversible inhibition of cell proliferation. Therefore, these three features were evaluated in order to assess the effect of resveratrol in CuSO_4_-SIPS fibroblasts. Briefly, cell proliferation was assessed by counting the viable cells at 0, 24, 48, and 72 h after copper removal. Then, at the last time-point (72 h), cell morphology was observed and the percentage of SA beta-gal positive cells was quantified for each condition. As shown in Figure 2(a), in the absence of resveratrol, CuSO_4_-SIPS fibroblasts presented the typical senescent morphology, as they were no longer small and fusiform and became enlarged and flattened. However, copper-treated cells recovering in the presence of resveratrol exhibited less pronounced senescent-like alterations, as they appeared thinner and more elongated, when compared to cells in the absence of resveratrol. This was particularly evident for the highest concentration of resveratrol used (10 *μ*M). It is noteworthy to mention that even cells not submitted to copper exhibited a slightly different aspect in the presence of resveratrol, as they seemed smaller and their cell limits were more clear-cut.

Similar to previously reported results [6], CuSO_4_-SIPS cellular model contained 34% of cells positive for SA beta-gal (Figure 2(b)), whereas the controls had only 5% of senescent cells. However, the addition of 5 and 10 *μ*M resveratrol to copper-treated cells resulted in a statistically significant reduction in the number of SA beta-gal positive cells (to 16 and 14%, resp.). The ability of copper sulfate to inhibit cell proliferation had previously been described [6] and herein is again demonstrated (Figure 2(c)), as 3 days after stress, copper-treated cells presented a reduction of 88% in their proliferation when compared to controls. Media supplementation with 5 and 10 *μ*M resveratrol during the recovery period resulted in the attenuation of cell proliferation inhibition by 20 and 34%, respectively. In addition, in the absence of copper, the selected concentrations of resveratrol were not able to affect significantly cell proliferation when compared to control cells. Altogether, these data show that resveratrol can actually attenuate the induction of senescence by copper sulfate in WI-38 fibroblasts.

### 3.3. Resveratrol Does Not Alter Copper-Induced Upregulation of Senescence-Associated Genes and Proteins

**Figure 3:**
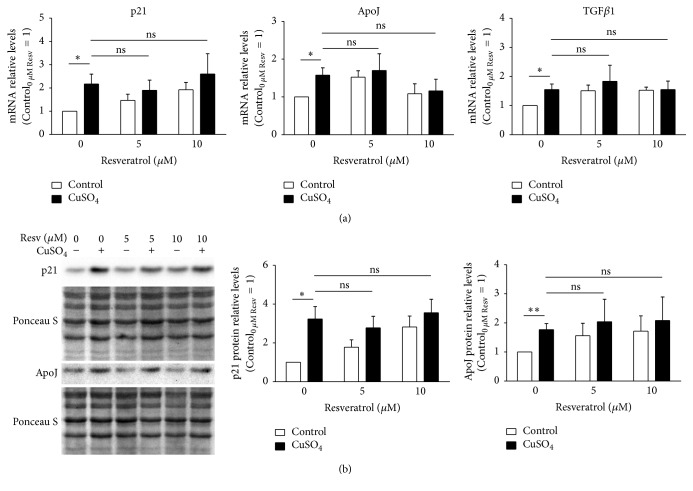
Resveratrol supplementation does not affect copper-induced expression of senescence-associated molecules. (a) Transcript relative levels of cyclin-dependent kinase inhibitor 1A (p21), apolipoprotein J (ApoJ), and transforming growth factor beta 1 (TGF*β*1) were assessed by qPCR in 350 *μ*M CuSO_4_-treated fibroblasts that were allowed to recover in the presence of the indicated doses of resveratrol. (b) Representative blots obtained for the determination of p21 and ApoJ protein level by western blot are depicted; the resulting densitometric analysis, normalized for control cells in the absence of resveratrol, is plotted for each analysed protein. Ponceau S staining was used to control protein loading. Data represent mean ± SEM of at least three independent experiments. ^*∗*^*p* < 0.05; ^*∗∗*^*p* < 0.01; and ^ns^non-significant, for the comparisons between the indicated groups.

There are several genes and proteins, such as the cyclin-dependent kinase inhibitor 1A (p21), apolipoprotein J (ApoJ), and transforming growth factor beta 1 (TGF*β*1), whose overexpression is typical of the senescent phenotype observed in RS and SIPS cellular models. Herein, we evaluated the ability of resveratrol to adjust the levels of p21, ApoJ, and TGF*β*1 upon copper treatment, in order to justify its effect in the attenuation of copper-induced senescence. Therefore, the mRNA transcript relative levels of these genes were quantified by qPCR (Figure 3(a)). In accordance with previous publication [5], p21, ApoJ, and TGF*β*1 mRNA levels were found upregulated by 2.2-, 1.6-, and 1.6-fold, respectively, in CuSO_4_-SIPS fibroblasts when compared to control cells. However, the addition of resveratrol (either 5 or 10 *μ*M) immediately after copper sulfate removal did not have any statistically significant effect on the transcript levels of these genes. To validate these results and exclude the occurrence of posttranslational regulation, the relative protein levels of p21 and ApoJ were evaluated by western blot (Figure 3(b)). At the protein level, p21 and ApoJ presented a 3.2- and 1.8-fold increase in copper-treated cells, when compared to controls, thus confirming the previously noticed trend. In addition, similarly to transcript levels results, resveratrol supplementation did not affect copper-induced augmentation of these proteins. Overall, the effect of resveratrol in the attenuation of copper-induced senescence does not involve the regulation of p21, ApoJ, and TGF*β*1 senescence-associated genes.

### 3.4. CuSO_4_-Induced Proteostasis Imbalance Is Attenuated by Resveratrol

**Figure 4:**
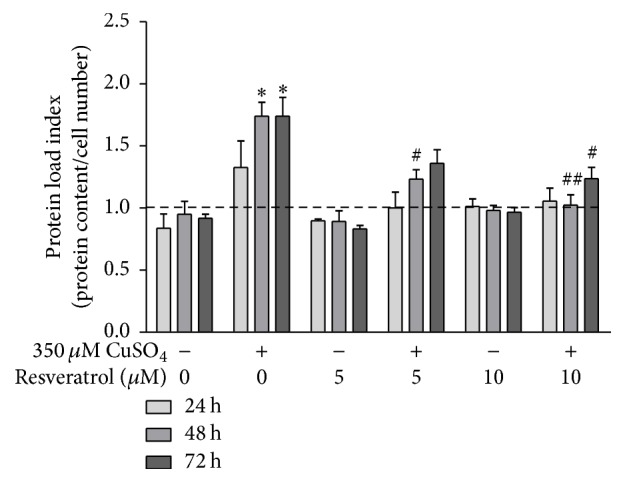
CuSO_4_-induced proteostasis imbalance is attenuated by resveratrol. Protein load index (PLI), used as a measure of cellular protein accumulation, was calculated as the ratio between total protein content and cell number for each condition, at different time-points after CuSO_4_ treatment (0, 24, 48, and 72 h). PLI values were normalized for the initial time-point (0 h) and the relative values are plotted for the indicated conditions. Data represent mean ± SEM of at least three independent experiments. ^*∗*^*p* < 0.05, when compared to control cells in the absence of resveratrol; ^#^*p* < 0.05 and ^##^*p* < 0.01, relatively to CuSO_4_-treated cells without resveratrol, at the respective time-points.

The occurrence of proteostasis imbalance is a major hallmark of aging [32] and, at the cellular level, may be shown by increased intracellular protein content [33]. To measure cellular protein accumulation for each experimental condition, the ratio between total protein content and cell number, here defined as the protein load index (PLI), was calculated at 0, 24, 48, and 72 h after CuSO_4_ removal (sodium sulfate for controls). Assuming that immediately after stress removal PLI equals 1, it was shown that it significantly increased 1.7-fold at 48 and 72 h time-points in CuSO_4_-SIPS cells when compared to the respective control conditions (Figure 4). CuSO_4_-treated fibroblasts that were allowed to recover in the presence of 5 *μ*M resveratrol exhibited a statistically significant 0.5-fold decrease in PLI at 48 h, when compared with cells without added resveratrol. Moreover, the addition of 10 *μ*M resveratrol after copper removal totally reverted PLI to the level of controls in the absence of copper at 48 h and presented a statistically significant 0.5-fold decrease at 72 h, when compared to copper-treated cells in the absence of resveratrol at the same time-point.

**Figure 5:**
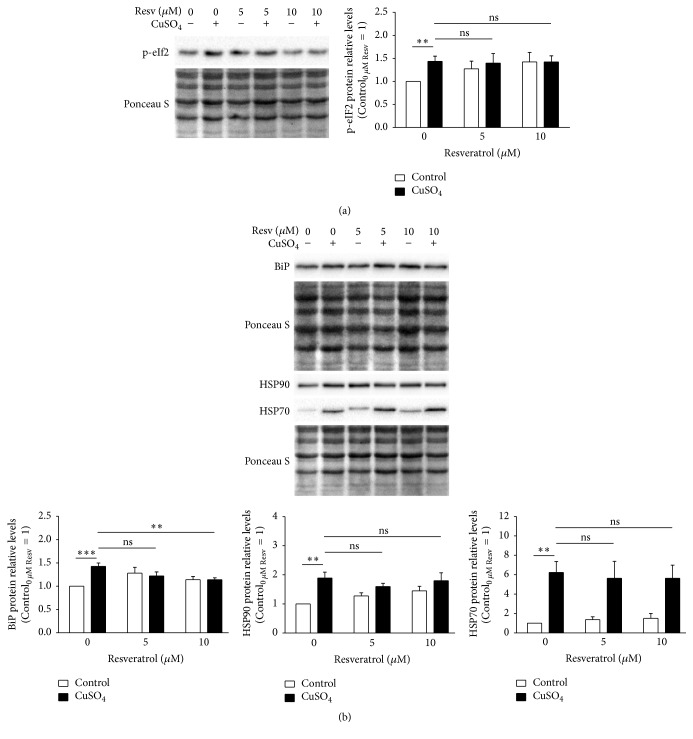
Resveratrol attenuates copper-induced BiP upregulation but has no effect on eIF2 phosphorylation or HSP90 and HSP70 expression. (a) Phosphorylated eukaryotic translation initiation factor 2 (p-eIF2) and (b) immunoglobulin binding protein (BiP), heat shock protein (HSP) 90, and HSP70 protein relative levels were determined by western blot at 72 h after the removal of 350 *μ*M CuSO_4_ (or Na_2_SO_4_, for controls) in fibroblasts that were allowed to recover in the presence or absence of resveratrol (5 or 10 *μ*M). Representative blots are depicted and densitometric quantification is plotted assuming that protein levels of each analysed protein in control cells without resveratrol equal 1. Ponceau S staining was used to normalize protein loading. Data represent mean ± SEM of at least three independent experiments. ^*∗∗*^*p* < 0.01; ^*∗∗∗*^*p* < 0.001; and ^ns^non-significant, for the comparisons between the indicated groups.

To compensate the altered proteostasis, CuSO_4_-SIPS cells present higher levels of phosphorylated eukaryotic translation initiation factor 2 (p-eIF2) [6], which inhibits general protein translation and allows cells to restore homeostasis. A possible explanation for the diminished PLI obtained for copper cells recovering in the presence of resveratrol could be an increased inhibition of overall protein synthesis caused by higher p-eIF2. When p-eIF2 was quantified by western blot (Figure 5(a)), as expected it was found increased in CuSO_4_-treated cells, compared to controls. However, resveratrol supplementation upon copper removal did not result in any additional alteration in p-eIF2 protein level. Next, cell chaperoning ability was evaluated by the quantification of the molecular chaperones immunoglobulin binding protein (BiP), heat shock protein (HSP) 90, and HSP70 by western blot (Figure 5(b)). In fact, the intracellular protein levels of BiP, HSP90, and HSP70 were 1.4-, 1.9-, and 6.3-fold increase in CuSO_4_-SIPS fibroblasts, when compared to control cells. The presence of resveratrol after copper removal had no effect on HSP90 and HSP70 protein levels comparing to the levels of copper-treated cells without resveratrol. However, BiP protein levels were diminished (to 1.1-fold) in copper-treated cells that were allowed to recover in the presence of 10 *μ*M resveratrol, relatively to the condition without resveratrol, reflecting a lower need to buffer defective or damaged proteins.

### 3.5. Resveratrol Attenuates CuSO_4_-Induced Accumulation of Modified Proteins by the Induction of Lysosomal Autophagy

**Figure 6:**
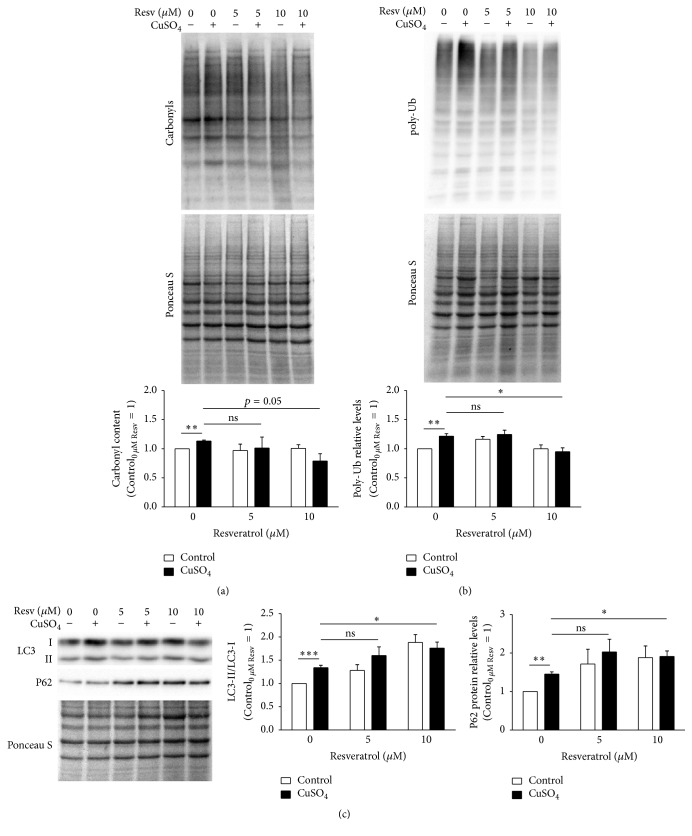
CuSO_4_-induced accumulation of carbonylated and polyubiquitinated proteins is reduced by resveratrol, through lysosomal autophagy induction. (a) Protein carbonyl content and (b) polyubiquitinated (poly-Ub) proteins were evaluated in fibroblasts submitted to the indicated conditions by western blot. Representative blots are depicted and densitometric quantification was normalized by attributing the value 1 for control cells in the absence of resveratrol. Ponceau S staining was used as protein loading control. (c) Lysosomal autophagy was studied by the conversion of LC3-I to LC3-II, a critical step for autophagosome formation, and quantification of P62, an ubiquitin-binding protein that target Ub-substrates to autophagosomes. LC3-II/LC3-I ratio and P62 relative levels were evaluated upon densitometric quantification and plotted assuming that control cells without resveratrol present a value of 1. Data represent mean ± SEM of at least three independent experiments. ^*∗*^*p* < 0.05; ^*∗∗*^*p* < 0.01; ^*∗∗∗*^*p* < 0.001; and ^ns^non-significant, for the comparisons between the indicated groups.

The altered proteostasis observed in CuSO_4_-SIPS fibroblasts could be a consequence of a progressive accumulation of oxidatively modified proteins. Protein carbonylation is a type of irreversible protein oxidation that is frequently used as an indicator of increased permanent levels of oxidative stress. Actually, cellular senescence models [34] and cells treated with oxidative stress inducers [35] were both shown to exhibit increased levels of carbonylated proteins. Herein, carbonyl protein content was evaluated to infer about cellular oxidative status in the different experimental conditions. CuSO_4_-SIPS cells presented a statistically significant 13% increase (*p* = 0.0017) in the relative levels of carbonylated proteins, when compared to control cells (Figure 6(a)). The addition of 10 *μ*M resveratrol during cell recovery (but not 5 *μ*M) was able to attenuate such increase in protein oxidation by 34%, a variation that was close to reach statistical significance (*p* = 0.054). These data suggest that resveratrol may be able to prevent or attenuate the accumulation of copper-induced oxidized proteins. This may be achieved either by its well described antioxidant properties that might prevent protein damage or by its ability to modulate protein degradation processes. UPS activity is known to be reduced during aging. The accumulation of polyubiquitinated (poly-Ub) proteins is usually associated with decreased UPS efficiency and, in fact, here a 22% increase in the levels of poly-Ub proteins in CuSO_4_-SIPS fibroblasts was observed (Figure 6(b)). In addition, resveratrol supplementation (only at 10 *μ*M) immediately after copper sulfate removal showed to be effective on restoring poly-Ub protein levels to the control cells ones, in a statistically significant manner (*p* = 0.026).

Depending on the conformation of the polyubiquitin chain that they possess, poly-Ub proteins may be degraded either in the proteasome or by lysosomal macroautophagy [36], mentioned as autophagy from here on in order to simplify. Autophagy plays a crucial role in the recycling of dysfunctional organelles and damaged protein aggregates and it was shown to be induced by resveratrol in order to prevent oxidative stress cellular damage [28, 29]. In the present study, the induction of autophagy was evaluated by the conversion of LC3-I to LC3-II, an essential step for autophagosome formation, by calculating the ratio of the LC3-II/LC3-I protein levels using western blot. Furthermore, the level of P62 protein, an ubiquitin-binding protein that serves as a link between LC3 and Ub substrates during autophagosome formation was also evaluated by western blot technique (Figure 6(c)). CuSO_4_-SIPS cells presented a statistically significant 1.4-fold increase in LC3-II/LC3-I ratio, when compared to young control fibroblasts. Furthermore, cell treatment with 10 *μ*M resveratrol after copper removal further increased this ratio (to 1.8-fold, *p* = 0.017), when compared to copper-treated cells that were allowed to recover in the absence of resveratrol. Accordingly, P62 protein levels were increased by 1.5-fold in CuSO_4_-SIPS, when compared to control. Moreover, exposure to 10 *μ*M resveratrol after copper removal resulted in additional increase in P62 protein expression (to 1.9-fold, *p* = 0.039).

## 4. Discussion

The CuSO_4_-SIPS cellular model has proven to have major value for studying molecular events that are responsible for the aging process [5, 6, 37]. Furthermore, it brought additional evidence supporting copper contribution to the age-related functional deterioration and to the progression of age-related disorders. The present study shows that CuSO_4_-induced cell senescence results in reduced Sirt1 expression. As Sirt1 is activated by the polyphenolic compound resveratrol, the mechanism and possibility of attenuating this senescent effect upon Sirt1 were addressed. In fact, it was demonstrated that resveratrol supplementation attenuated copper-induced appearance of some typical senescence features. In addition, the mechanism behind such antisenescence effect of resveratrol was shown to involve the modulation of cellular proteostasis either by the protection of proteins from oxidative damage or by the induction of protein degradation processes.

The effect of resveratrol on cellular senescence has been investigated, but the results are contradictory: while some authors reported resveratrol ability to attenuate cellular aging [12–14], others showed that it induced the appearance of senescence [38–41]. In either case, the molecular mechanisms involved in such effects were not fully clear. We believe that this discrepancy is explained by the different experimental conditions utilized in these studies: resveratrol ability to induce cell senescence was often reported using tumor cell lines [38–40] treated with high concentrations of the compound (above 25 *μ*M) that in some cases resulted in pro-apoptotic effects [41]; in turn, antiaging effects were described in nontumor cell lines, incubated with lower doses of resveratrol [12]. In line with these evidences, herein, the administration of 5 or 10 *μ*M resveratrol immediately after copper sulfate removal was able to attenuate the induction of WI-38 fibroblast cellular senescence, as the percentage of SA beta-gal positive cells was decreased, the typical morphological alterations were less evident, and the blockage of cell cycle was alleviated. However, in this study, resveratrol was not able to attenuate copper-induced upregulation of senescence-associated molecules, such as p21, ApoJ, and TGF*β*1. This indicates that the mechanism behind the positive antisenescence effects of resveratrol does not involve the inhibition of copper-induced expression of such senescence-associated genes.

It was recently reported that both RS and CuSO_4_-SIPS models exhibit altered expression of several ER molecular chaperones and enzymes and activated ER UPR pathways [6]. Here, CuSO_4_-SIPS fibroblasts exhibited greater total protein content, as measured by augmented PLI, increased expression of BiP, HSP70, and HSP90 molecular chaperones, a rise on the levels of carbonylated proteins, and higher amount of polyubiquitinated proteins, adding further evidence to the occurrence of proteostasis disruption during senescence. Nevertheless, our hypothesis that increased PLI reflects impaired proteostasis could be further supported by experimental evidence obtained for instance after inhibiting protein degradation mechanisms such as autophagy or the UPS. At present, the actual underlying molecular conditions triggering PLI increase are still unknown, but involvement of the typical cell enlargement associated with the senescence phenotype, or other mechanisms apart from proteostasis disruption, cannot be excluded. CuSO_4_-SIPS fibroblasts that were allowed to recover in the presence of resveratrol presented improved cellular proteostasis, as their total protein levels were similar to controls, BiP chaperone expression was attenuated, and poly-ubiquitinated proteins level was reduced. Altogether, these data demonstrated that, in the presence of resveratrol, cells were able to circumvent copper-induced disruption of cellular proteostasis, intimately related to the appearance of the typical senescent phenotype.

The well documented antioxidant properties of resveratrol are the likely contributors to this cell proteostasis maintenance effect, as it can protect proteins from being oxidized in a concentration and time-dependent manner. In fact, using in vitro oxidative stressed erythrocytes, resveratrol ability to prevent protein oxidation reaches a maximum protective effect between 30 and 60 minutes after polyphenolic compound addition and is then slightly reduced with time [42]. In the current study, resveratrol supplementation for 72 h attenuated the amount of carbonylated proteins on copper-treated cells in a variation that was close to reach statistical significance. A time-course evaluation of protein carbonylation along these 72 h should add further information on the existence of time-dependent variations on resveratrol efficiency to protect proteins from oxidation.

Another important resveratrol contribution for the modulation of cellular proteostasis is its ability to regulate protein degradation mechanisms, such as the UPS [26, 27] or lysosomal autophagy [28, 29]. Both mechanisms were shown to be intimately related as autophagy is activated to compensate UPS inhibition [43]. In brief, autophagy is crucial to degrade dysfunctional organelles and damaged protein aggregates and involves the formation of autophagosomes that are targeted to lysosomes for the degradation of their inner content. Autophagosome formation occurs in successive stages that depend on the concerted action of several proteins [44]. The cytosolic soluble protein LC3-I is particularly important in this process because it is lipidated to originate LC3-II, which integrates the autophagosome membrane. As such conversion is essential for elongation and maturation of the autophagosomes, LC3-II/LC3-I ratio is usually used to detect autophagy activation. In addition, as P62 protein is crucial to target poly-Ub-substrates into autophagosomes through LC3 binding [44], its detection further indicates such activation. Here, CuSO_4_-SIPS cells exhibited an increase both in LC3-I to LC3-II conversion and in P62 protein levels; when allowed to recover in the presence of resveratrol, LC3-II/LC3-I ratio and P62 protein levels were even higher, indicating an enhanced induction of autophagy and targeting of poly-Ub-substrates to autophagy. These results are in agreement with previous in vitro [45] and in vivo [28] studies demonstrating that oxidative stress conditions promote LC3-II/LC3-I ratio increase, further enhanced in the presence of resveratrol. Moreover, resveratrol has recently been described as able to promote the flux of proteins through the autophagosomal-lysosomal pathway, thus attenuating the dysfunctional effect of intracellular accumulation of damaged or defective proteins [27]. This promotion is in agreement with the results of the current study that favor resveratrol antisenescence effect as a consequence of its ability to improve cellular proteostasis through autophagy induction. However, the present study has some limitations regarding the actual induction of autophagy by resveratrol; further functional studies monitoring autophagosome number and the autophagic flux [46] in the presence of resveratrol, would clarify its effect on such processes. Moreover, given the proven crosstalk between autophagy and proteasomal degradation [47], we cannot exclude, in addition, the beneficial effects resulting from resveratrol ability to modulate the ubiquitin-proteasome system.

## 5. Conclusions

This study demonstrates that resveratrol is able to attenuate the induction of cell senescence resulting from CuSO_4_ exposure. Such effects result from resveratrol ability to promote cellular adaptive mechanisms, as autophagy upregulation, which sustain cellular proteostasis and confer cellular resistance to stress. Cellular proteostasis maintenance was found to be crucial to prevent the development of the senescent phenotype. These data also uncover molecular targets whose modulation is likely to prevent age-associated cell and tissue function deterioration and improve human healthspan.
