# Erratum to “Resveratrol Attenuates Copper-Induced Senescence by Improving Cellular Proteostasis”

**DOI:** 10.1155/2017/9172085

**Published:** 2017-09-24

**Authors:** Liliana Matos, Alexandra Monteiro Gouveia, Henrique Almeida

**Affiliations:** ^1^Departamento de Biologia Experimental, Faculdade de Medicina, IBMC, Instituto de Biologia Molecular e Celular and I3S, Instituto de Investigação e Inovação em Saúde, Universidade do Porto, Alameda Prof. Hernâni Monteiro, 4200-319 Porto, Portugal; ^2^Faculdade de Ciências da Nutrição e Alimentação, Universidade do Porto, Rua Dr. Roberto Frias, 4200-465 Porto, Portugal

In the article titled “Resveratrol Attenuates Copper-Induced Senescence by Improving Cellular Proteostasis” [1], there were a number of language errors, due to publisher error. The corrected version of the article is shown below:

## Abstract

Copper sulfate-induced premature senescence (CuSO_4_-SIPS) consistently mimics molecular mechanisms of replicative senescence, particularly at the endoplasmic reticulum proteostasis level. In fact, disruption of protein homeostasis has been associated with age-related cell/tissue dysfunction and susceptibility to human disorders. Resveratrol is a polyphenolic compound with proven antiaging properties under certain conditions. In this setting, we aimed to evaluate the ability of resveratrol to attenuate cellular senescence induction and to unravel related molecular mechanisms. Resveratrol attenuated typical senescence-induced alterations to cell morphology, senescence-associated beta-galactosidase activity, and cell proliferation in CuSO_4_-SIPS WI-38 fibroblasts. The mechanisms implicated in this antisenescence effect seem to be independent of the regulation of senescence-associated genes and proteins but are reliant on cellular proteostasis improvement. In fact, resveratrol supplementation restores copper-induced increases in protein content, attenuates immunoglobulin-binding protein levels, and reduces carbonylated and polyubiquitinated proteins by inducing autophagy. Our data provide compelling evidence for the beneficial effects of resveratrol by mitigating stressful consequences associated with CuSO_4_-SIPS via modulation of protein quality control systems. These findings highlight the importance of balanced cellular proteostasis and add further knowledge regarding molecular mechanisms mediating the antisenescence effects of resveratrol. Moreover, they contribute to the identification of specific molecular targets whose modulation may prevent age-associated cell dysfunction and improve human healthspan.

## 1. Introduction

Normal somatic dividing cells have been proven to be a valuable in vitro model to study cellular senescence and unravel molecular mechanisms and pathways implicated in the human aging process. The well-known model of replicative senescence (RS) is achieved when human diploid fibroblasts (HDFs) spontaneously stop dividing after an initial active period of population doublings (PDs) and become unresponsive to mitogenic stimuli [1]. In addition to irreversible cell cycle arrest, RS fibroblasts exhibit other typical, morphological, and molecular features, such as increased cellular volume, higher senescence-associated beta-galactosidase (SA beta-gal) activity, and increased expression of senescence-associated genes and proteins [2, 3]. A similar senescent phenotype, termed stress-induced premature senescence (SIPS), can be attained by exposing HDFs to subcytotoxic doses of oxidative stress inducers, such as hydrogen peroxide (H_2_O_2_-SIPS) [4], tert-butyl hydroperoxide, ultraviolet B radiation [3], or copper sulfate (CuSO_4_-SIPS) [5]. Recently, the latter was shown to mimic the RS model better than the most frequently used H_2_O_2_-SIPS model [6].

Resveratrol is a natural polyphenolic compound that has been shown to increase the maximum lifespan of several organisms, such as* Saccharomyces cerevisiae *[7],* Caenorhabditis elegans *[8],* Drosophila melanogaster* [9], and the short-lived fish* Nothobranchius furzeri *[10]. Yet, resveratrol failed to extend longevity in rodent mammals even though it improved their healthspan, thus providing evidence for a protective role against age-related deterioration [11].

At the cellular level, resveratrol has been shown to attenuate senescent features in both RS [12] and H_2_O_2_-SIPS [13, 14] cellular models. These antiaging effects have long been associated with the ability of resveratrol to activate sirtuin 1 (Sirt1) deacetylase [15]. Further, it has been demonstrated that Sirt1 overexpression attenuates senescence and extends the replicative lifespan of several cultured cell types [16–18], while its inhibition results in increased cellular senescence [16]. Downregulation of Sirt1 has been associated with aging [19] and has been observed in cellular senescence models [20, 21], further demonstrating its preventive role in the features of senescence. Besides the ability of resveratrol to modulate signal transduction pathways via activation of Sirt1 [14, 22], several other biological events have been assigned to be responsible for its positive effects, including its ability to increase stress resistance [12], induce telomerase activity [23], decrease the secretion of senescence-associated proinflammatory proteins [24], and inhibit the mechanistic target of rapamycin (mTOR) [13]. Resveratrol has also been found to modulate protein quality control cellular responses, as it regulates the expression of heat shock molecular chaperones [25] and promotes cellular protein degradation mechanisms, namely, the ubiquitin-proteasome system (UPS) [26, 27] and lysosomal autophagy [28, 29]. Moreover, resveratrol was able to increase the lifespan of* C. elegans* via upregulation of activated in blocked unfolded protein response-11* (abu11)*, which encodes a protein involved in the endoplasmic reticulum (ER) unfolded protein response (UPR) that protects organisms from damage by improperly folded proteins [8].

In the present study, we aimed to evaluate the ability of resveratrol to attenuate the establishment of cellular senescence upon CuSO_4_ induction, unraveling the molecular mechanisms that might be involved. We found that resveratrol supplementation was able to reduce the appearance of some senescence-associated features by improving cellular proteostasis, likely via prevention of oxidative damage to proteins and the induction of protein degradation mechanisms, which prevent the accumulation of damaged proteins.

## 2. Material and Methods

### 2.1. Cell Culture

WI-38 human fetal lung fibroblasts were purchased from The European Collection of Cell Cultures (ECACC) and were cultivated in complete Basal Medium Eagle (BME) supplemented with 10% fetal bovine serum at 37°C in a 5% CO_2_ humidified atmosphere. WI-38 cells are young, with less than 30 PDs, and enter senescence at 45 PDs or above. For the induction of CuSO_4_-SIPS, subconfluent young WI-38 fibroblasts were exposed to 350 *μ*M CuSO_4_ (Na_2_SO_4_ for controls) for 24 h. Cells were then washed once with phosphate buffered saline (PBS) and the medium was replaced with fresh complete medium containing 5 or 10 *μ*M resveratrol (R5010-Sigma-Aldrich®) for an additional 72 h. Control cells were exposed to a final concentration of 0.1% dimethyl sulfoxide (DMSO) for the same period.

### 2.2. Cell Morphology and SA beta-Gal Detection

Cell morphology evaluation was performed 72 h after copper removal via optical inspection with an inverted microscope. To assess the presence of senescent cells, SA beta-gal was detected 72 h after copper removal as previously described [5]. The percentage of SA beta-gal-positive cells in each condition was determined by microscopically counting 400 total cells/well from at least three independent experiments.

### 2.3. Cell Proliferation and Total Protein Content

 To assess the effects of the different treatments on cell proliferation and total protein content, cell numbers were determined and a sulforhodamine B (SRB) assay [30] was performed over time after copper removal. Briefly, 3000 cells/well were seeded in 96-well culture plates, treated for 24 h with CuSO_4_ (or Na_2_SO_4_ for controls), and then analyzed at different time points (0, 24, 48, and 72 h) while recovering in the presence or absence of resveratrol. For cell number determination, cells were trypsinized and stained with trypan blue; viable cells were microscopically counted in a Neubauer chamber. The total number of cells per well for each condition at the different time points was calculated and plotted; at *t* = 0 for each condition, cell numbers were assumed to equal 1. To determine total protein content, cells were treated with 10% trichloroacetic acid (TCA) for 1 h at 4°C. The TCA-precipitated proteins fixed at the bottom of the wells were stained for 30 minutes with 0.057% (w/v) SRB in a 1% acetic acid solution and then washed four times with 1% acetic acid. Bound dye was solubilized with 10 mM Tris base solution, and absorbance at 510 nm for each well was recorded using a microplate reader (Infinite 200, Tecan).

### 2.4. Real Time PCR

*Gene* expression experiments were performed 72 h after CuSO_4_ treatment via real time quantitative PCR (qPCR). Total RNA, extracted (PureLink® RNA Mini Kit, Ambion) from cells derived from at least three independent cultures for each condition, was converted into cDNA via reverse transcription. Amplification reaction assays contained SYBR Green Mastermix (SYBR® Select Master Mix, Applied Biosystems®), 50 ng cDNA, and primers (STAB VIDA, Lda.) at optimal concentrations. The primer sequences were p21, 5′-CTGGAGACTCTCAGGGTCGAA-3′ and 5′-CCAGGACTGCAGGCTTCCT-3′; ApoJ, 5′-GGATGAAGGACCAGTGTGACAAG-3′ and 5′-CAGCGACCTGGAGGGATTC-3′; TGF*β*1, 5′-AGGGCTACCATGCCAACTTCT-3′ and 5′-CCGGGTTATGCTGGTTGTACA-3′; and TATA box binding protein (TBP), 5′-TCAAACCCAGAATTGTTCTCCTTAT-3′ and 5′-CCTGAATCCCTTTAGAATAGGGTAGA-3′. The protocol used for qPCR was 95°C (3 min), 40 cycles of 95°C (15 sec), and 60°C (1 min). qPCR was performed in a StepOnePlus™ thermal cycler (Applied Biosystems™). TBP was the selected housekeeping gene when calculating relative transcript levels of the target genes.

### 2.5. Western Blot

 Protein levels were assessed 72 h after CuSO_4_ exposure by western blot analysis. WI-38 cells exposed to the different treatments were washed with PBS and scraped on ice in a lysis buffer (10 mM Tris, pH 7.4; 100 mM NaCl; 1 mM EDTA; and 0.1% Triton X-100) supplemented with a protease inhibitor cocktail (Sigma-Aldrich). After the Bradford assay was conducted, 20 *μ*g (or 10 *μ*g for the detection of carbonylated or polyubiquitinated (poly-Ub) proteins) protein from each cell extract was resolved by sodium dodecyl sulfate polyacrylamide gel electrophoresis (SDS-PAGE). Proteins were blotted into a nitrocellulose membrane and, after blocking with 5% nonfat dry milk diluted in Tris-buffered saline 0.1% tween 20 (TBST), probed with specific primary antibodies (anti-HSP90 ab13495 and anti-p62 ab109012, Abcam®; anti-LC3 NB100-2220, Novus Biologicals; anti-ubiquitin PW0930, Enzo® Life Sciences; anti-p21 #2946, anti-phospho-eukaryotic translation initiation factor 2 [p-eIF2] #3398, anti-HSP70 #4876, and anti-BiP #3177, Cell Signaling Technology®) overnight at predetermined optimal dilutions. For the specific detection of carbonylated proteins, immediately after protein transfer, the nitrocellulose-bound proteins were treated as described elsewhere [31]. Briefly, the membranes were equilibrated in 20% methanol in TBS, washed for 5 min with 10% trifluoroacetic acid (TFA), derivatized with 5 mM 2,4-dinitrophenylhydrazine (DNPH, Sigma-Aldrich) diluted in 10% TFA for 10 min (protected from light), washed with 10% TFA to remove the excess DNPH, and finally washed with 50% methanol. Following this procedure, the membranes were blocked with 5% bovine serum albumin in TBST and incubated with primary anti-DNP antibodies (D9656, Sigma-Aldrich). After this point, the western blot procedure was similar for all antibodies. Specifically, after TBST washing, immunoblots were incubated with the appropriate peroxidase-conjugated secondary antibodies for 1 h, detected using ECL western blotting substrate (Pierce™, Thermo Scientific), and visualized in a ChemiDocTM XRS (BioRad Laboratories). Results were quantified by densitometry using Image Lab® software. Protein loading was normalized using Ponceau S protein staining; however, similar data were obtained when protein loading was normalized to tubulin (data not shown).

### 2.6. Statistical Analysis

Student's *t*-test was used to compare the means between two different conditions. A *p* value lower than 0.05 was considered statistically significant.

## 3. Results

### 3.1. Sirt1 Expression Is Diminished in CuSO_4_-SIPS

 Previously, it was demonstrated that Sirt1 expression decreases with increasing PDs [20] and in H_2_O_2_-SIPS cellular models [21]. Here, Sirt1 mRNA and protein levels were evaluated by qPCR and western blot, respectively, in CuSO_4_-induced senescent WI-38 fibroblasts. Similarly to other RS and SIPS models, gene (Figure 1(a)) and protein (Figure 1(b)) expression of Sirt1 decreased in CuSO_4_-SIPS fibroblasts. Namely, mRNA and relative protein levels were 27% and 23% lower, respectively, in copper-treated cells than in controls (*p* = 0.04 and *p* = 0.008, resp.). The effects of resveratrol (5 or 10 *μ*M), a Sirt1 activator, were evaluated 72 h after incubating the cells with CuSO_4_ for 24 h, which is the usual recovery time that cells need to adapt and develop the senescent phenotype [5]. The addition of 10 *μ*M resveratrol attenuated copper-induced decrease in Sirt1 protein levels (*p* = 0.047) to values similar to those observed in young control cells. Sirt1 protein levels were 1.6- and 1.3-fold (*p* = 0.008 and *p* = 0.01) higher in non-CuSO_4_ exposed fibroblasts incubated with 5 and 10 *μ*M resveratrol for 72 h, respectively, than in young control cells (Figure 1(b)).

### 3.2. Resveratrol Attenuates the Appearance of Some Typical Senescence-Associated Alterations

Senescent cells usually present typical morphological alterations, increased levels of SA beta-gal, and irreversible inhibition of cell proliferation. Therefore, these three features were evaluated to assess the effects of resveratrol in CuSO_4_-SIPS fibroblasts. Briefly, cell proliferation was assessed by counting viable cells 0, 24, 48, and 72 h after copper removal. Cell morphology was observed at the final time point (72 h), and the percentage of SA beta-gal-positive cells was quantified for each condition. As shown in Figure 2(a), CuSO_4_-SIPS fibroblasts presented with typical senescent morphology in the absence of resveratrol, as they were enlarged and flattened rather than long, small, and fusiform. However, copper-treated cells recovering in the presence of resveratrol exhibited less pronounced senescent-like alterations, as they appeared thinner and more elongated than cells recovering in the absence of resveratrol. This phenomenon was particularly evident for the highest concentration of resveratrol used (10 *μ*M). It is worth mentioning that slightly different morphological aspects were observed in cells that were not exposed to copper but were treated with resveratrol; specifically, they appeared smaller and their cell limits were more clear-cut.

Similar to previously reported results [6], 34% of cells were positive for SA beta-gal in the CuSO_4_-SIPS cellular model (Figure 2(b)), whereas only 5% of control cells were senescent. However, the addition of 5 and 10 *μ*M resveratrol to copper-treated cells resulted in a statistically significant reduction in the number of SA beta-gal positive cells (to 16 and 14%, resp.). The ability of CuSO_4_ to inhibit cell proliferation had previously been described [6] and is again demonstrated in this study (Figure 2(c)); 3 days after stress, proliferation was 88% lower in copper-treated cells than in controls. Supplementation of the media with 5 and 10 *μ*M resveratrol during the recovery period resulted in the attenuation of cell proliferation inhibition by 20 and 34%, respectively. In addition, cell proliferation did not differ significantly in the absence of copper from that in the control cells for selected concentrations of resveratrol. Altogether, these data show that resveratrol attenuates the induction of senescence by CuSO_4_ in WI-38 fibroblasts.

### 3.3. Resveratrol Does Not Alter Copper-Induced Upregulation of Senescence-Associated Genes and Proteins

There are several genes and proteins, such as p21, ApoJ, and TGF*β*1, whose overexpression is typical of the senescent phenotype observed in RS and SIPS cellular models. Herein, we evaluated the ability of resveratrol to modulate the levels of p21, ApoJ, and TGF*β*1 upon copper treatment to explain its ability to attenuate copper-induced senescence. Therefore, the relative mRNA transcripts of these genes were quantified by qPCR (Figure 3(a)). In accordance with a previous publication [5], p21, ApoJ, and TGF*β*1 mRNA levels were 2.2-, 1.6-, and 1.6-fold higher, respectively, in CuSO_4_-SIPS fibroblasts than in control cells. However, the addition of resveratrol (either 5 or 10 *μ*M) immediately after removal of the CuSO_4_ did not have any statistically significant effects on the transcript level of these genes. To validate these results and exclude the occurrence of posttranslational regulation, the relative protein levels of p21 and ApoJ were evaluated by western blot (Figure 3(b)). At the protein level, p21 and ApoJ were 3.2- and 1.8-fold higher in copper-treated cells than in controls, thus confirming the previously observed trend. In addition, similar to the mRNA transcript results, resveratrol supplementation did not affect copper-induced augmentation of these proteins. Overall, resveratrol-associated attenuation of copper-induced senescence does not involve the regulation of p21, ApoJ, and TGF*β*1 senescence-associated genes.

### 3.4. CuSO_4_-Induced Proteostasis Imbalance Is Attenuated by Resveratrol

 A proteostasis imbalance is a major hallmark of aging [32] and has been demonstrated at the cellular level by increased intracellular protein content [33]. To measure cellular protein accumulation for each experimental condition, the ratio between total protein content and cell number, here defined as the protein load index (PLI), was calculated 0, 24, 48, and 72 h after CuSO_4_ removal (sodium sulfate for controls). Assuming that the PLI equals 1 immediately after stress removal, PLI values were 1.7-fold higher in CuSO_4_-SIPS cells at 48 and 72 h than in the respective control cells (Figure 4). CuSO_4_-treated fibroblasts that were allowed to recover in the presence of 5 *μ*M resveratrol had PLI values that were statistically lower (0.5-fold) at 48 h than cells without added resveratrol. Moreover, the addition of 10 *μ*M resveratrol after copper removal totally reverted the PLI values to those observed in controls in the absence of copper at 48 h, and the PLI values were significantly lower (0.5-fold) at 72 h than those in copper-treated cells in the absence of resveratrol at the same time point.

To compensate for the altered proteostasis, CuSO_4_-SIPS cells present higher levels of p-eIF2 [6], which inhibits general protein translation and allows cells to restore homeostasis. A possible explanation for the diminished PLI obtained for copper-treated cells recovering in the presence of resveratrol could be an increase in the inhibition of overall protein synthesis caused by higher p-eIF2. As expected, p-eIF2 levels were higher in CuSO_4_-treated cells than in control cells, as determined by western blot (Figure 5(a)). However, resveratrol supplementation upon copper removal did not result in any additional alterations in p-eIF2 protein levels. Next, cell chaperoning ability was evaluated by the quantification of BiP, HSP90, and HSP70 by western blot (Figure 5(b)). The intracellular protein levels of BiP, HSP90, and HSP70 were 1.4-, 1.9-, and 6.3-fold higher in CuSO_4_-SIPS fibroblasts than in control cells. The presence of resveratrol after copper removal had no effect on HSP90 and HSP70 protein levels compared to the levels of copper-treated cells without resveratrol. However, BiP protein levels diminished (to 1.1-fold) in copper-treated cells that were allowed to recover in the presence of 10 *μ*M resveratrol relative to the condition without resveratrol, reflecting a lesser need to buffer defective or damaged proteins.

### 3.5. Resveratrol Attenuates CuSO_4_-Induced Accumulation of Modified Proteins by the Induction of Lysosomal Autophagy

The altered proteostasis observed in CuSO_4_-SIPS fibroblasts could be a consequence of a progressive accumulation of oxidatively modified proteins. Protein carbonylation is a type of irreversible protein oxidation that frequently serves as an indicator of increased permanent levels of oxidative stress. Moreover, cellular senescence models [34] and cells treated with oxidative stress inducers [35] were both shown to exhibit increased levels of carbonylated proteins. Herein, carbonyl protein content was evaluated to infer the cellular oxidative status under the different experimental conditions. CuSO_4_-SIPS cells showed a 13% increase (*p* = 0.0017) in the relative levels of carbonylated proteins, when compared to control cells (Figure 6(a)). The addition of 10 *μ*M, but not 5 *μ*M, resveratrol during cell recovery attenuated the increase in protein oxidation by 34%, a result that was close to reaching statistical significance (*p* = 0.054). These data suggest that resveratrol prevents or attenuates the accumulation of copper-induced oxidation of proteins. This may be achieved either by its well-described antioxidant properties, which might prevent protein damage, or by its ability to modulate protein degradation processes. UPS activity is known to be reduced during aging. The accumulation of poly-Ub proteins is usually associated with decreased UPS efficiency. A 22% increase in the levels of poly-Ub proteins in CuSO_4_-SIPS fibroblasts was observed in this study (Figure 6(b)). In addition, supplementation with resveratrol (at 10 *μ*M only) immediately after CuSO_4_ removal was effective for restoring poly-Ub protein levels to those observed in the control cells in a statistically significant manner (*p* = 0.026).

Depending on the conformation of their polyubiquitin chains, poly-Ub proteins may be degraded either in the proteasome or by lysosomal macroautophagy [36] (termed autophagy from now on for simplicity). Autophagy plays a crucial role in the recycling of dysfunctional organelles and damaged protein aggregates, and it was shown to be induced by resveratrol in order to prevent cellular damage from oxidative stress [28, 29]. In the present study, the induction of autophagy was evaluated by calculating the ratio of LC3-II/LC3-I proteins via western blot, which represents the conversion of LC3-I to LC3-II, an essential step for autophagosome formation. Furthermore, the level of P62 protein, a ubiquitin-binding protein that serves as a link between LC3 and Ub substrates during autophagosome formation, was also evaluated by western blot (Figure 6(c)). CuSO_4_-SIPS cells presented a statistically significant 1.4-fold increase in LC3-II/LC3-I ratio, when compared to young control fibroblasts. Furthermore, this ratio was further increased to levels that were 1.8-fold higher (*p* = 0.017) in cells treated with 10 *μ*M resveratrol than in copper-treated cells that were allowed to recover in the absence of resveratrol. Accordingly, P62 protein levels increased 1.5-fold in CuSO_4_-SIPS compared to the control cells. Moreover, exposure to 10 *μ*M resveratrol after copper removal resulted in an additional increase in P62 protein expression (to 1.9-fold, *p* = 0.039).

## 4. Discussion

The CuSO_4_-SIPS cellular model has proven to have major value for studying molecular events that are responsible for the aging process [5, 6, 37]. Furthermore, it provides additional evidence supporting the contribution of copper to age-related functional deterioration and the progression of age-related disorders. The present study shows that CuSO_4_-induced cell senescence results in reduced Sirt1 expression. As Sirt1 is activated by the polyphenolic compound, resveratrol, the mechanisms and possibility of attenuating this senescent effect via Sirt1 were addressed. We demonstrated that resveratrol supplementation attenuates the copper-induced appearance of some typical features of senescence. In addition, the mechanisms underlying such antisenescence effects of resveratrol involve the modulation of cellular proteostasis, via either protection of proteins from oxidative damage or the induction of protein degradation processes.

The effects of resveratrol on cellular senescence have been investigated; however, the results are contradictory. Specifically, some authors have reported the ability of resveratrol to attenuate cellular aging [12–14], whereas others have shown that it induces senescence [38–41]. In either case, the molecular mechanisms involved in such effects are not fully clear. We believe that these discrepancies can be attributed to the different experimental conditions utilized in these studies. The ability of resveratrol to induce cell senescence is often reported in studies using tumor cell lines [38–40] treated with high concentrations of the compound (above 25 *μ*M), which in some cases results in proapoptotic effects [41]; in contrast, antiaging effects are described in nontumor cell lines incubated with lower doses of resveratrol [12]. In support of these data, administration of 5 or 10 *μ*M resveratrol immediately after CuSO_4_ removal attenuated the induction of WI-38 fibroblast cellular senescence in this study, as the percentage of SA beta-gal-positive cells decreased, the typical morphological alterations were less evident, and blockage of the cell cycle was alleviated. However, in this study, resveratrol did not attenuate copper-induced upregulation of senescence-associated molecules such as p21, ApoJ, and TGF*β*1. These results indicate that the mechanisms underlying the positive antisenescence effects of resveratrol do not involve inhibition of the copper-induced expression of senescence-associated genes.

Recently, it was reported that both RS and CuSO_4_-SIPS models exhibit altered expression of several ER molecular chaperones and enzymes and activated ER UPR pathways [6]. Here, CuSO_4_-SIPS fibroblasts exhibited greater total protein content, as determined by augmented PLI values; increased expression of BiP, HSP70, and HSP90 molecular chaperones; a rise in the levels of carbonylated proteins; and more poly-Ub proteins, adding further evidence to support the occurrence of proteostasis disruption during senescence. Nevertheless, our hypothesis that increased PLI values reflect impaired proteostasis could be further supported by additional experimental evidence such as the inhibition of protein degradation mechanisms, including autophagy or UPS. At present, the underlying molecular conditions that trigger increases in PLI values are still unknown; however, the typical enlargement of the cell, which is associated with the senescence phenotype, or other mechanisms apart from proteostasis disruption cannot be excluded. CuSO_4_-SIPS fibroblasts that were allowed to recover in the presence of resveratrol showed improved cellular proteostasis, as their total protein levels were similar to those in controls, BiP chaperone expression was attenuated, and poly-Ub protein levels were reduced. Altogether, these data demonstrate that, in the presence of resveratrol, cells can circumvent copper-induced disruption of cellular proteostasis, which is intimately related to the appearance of the typical senescent phenotype.

The well-documented antioxidant properties of resveratrol are the likely contributors to the cell proteostasis-maintenance effect reported here, as resveratrol is known to protect proteins from becoming oxidized in a concentration- and time-dependent manner. In fact, using in vitro oxidative-stressed erythrocytes, resveratrol prevented protein oxidation, reaching a maximum protective effect between 30 and 60 min after its addition; this phenomenon was slightly reduced over time [42]. In the current study, resveratrol supplementation for 72 h attenuated the amount of carbonylated proteins in copper-treated cells, an effect that was close to reaching statistical significance. A time-course evaluation of protein carbonylation for 72 h would add further information regarding the existence of time-dependent variations in the ability of resveratrol to protect proteins from oxidation.

Another important resveratrol contribution for the modulation of cellular proteostasis is its ability to regulate protein degradation mechanisms, such as UPS [26, 27] or lysosomal autophagy [28, 29]. Both mechanisms have been shown to be intimately related, as autophagy is activated to compensate for UPS inhibition [43]. In brief, autophagy is crucial for degrading dysfunctional organelles and damaged protein aggregates and involves the formation of autophagosomes that are targeted to lysosomes for the degradation of their inner content. Autophagosome formation occurs in successive stages that depend on the concerted action of several proteins [44]. The cytosolic soluble protein, LC3-I, is particularly important in this process because it is lipidated to form LC3-II, which integrates the autophagosome membrane. As such, conversion is essential for elongation and maturation of autophagosomes; the LC3-II/LC3-I ratio is usually used to detect autophagy activation. In addition, the P62 protein is crucial for targeting poly-Ub substrates to autophagosomes via LC3 binding [44], and its detection further indicates such activation. Here, CuSO_4_-SIPS cells exhibited an increase in LC3-I to LC3-II conversion and P62 protein levels; when they were allowed to recover in the presence of resveratrol, the LC3-II/LC3-I ratio and P62 protein levels were even higher, indicating an enhanced induction of autophagy and targeting of poly-Ub substrates to the autophagosome. These results agree with previous in vitro [45] and in vivo [28] studies demonstrating that oxidative stress promotes increases in the LC3-II/LC3-I ratio, which is further enhanced in the presence of resveratrol. Moreover, it was recently shown that resveratrol promotes the flux of proteins through the autophagosomal-lysosomal pathway, thus attenuating the dysfunctional effects caused by the intracellular accumulation of damaged or defective proteins [27]. These results are concordant with those of the current study, favoring resveratrol antisenescence effects because of improved cellular proteostasis via autophagy induction. However, the present study has some limitations regarding the actual induction of autophagy by resveratrol, and further functional studies monitoring autophagosome number and the autophagic flux [46] in the presence of resveratrol would clarify its effect on such processes. Moreover, given the proven crosstalk between autophagy and proteasomal degradation [47], we cannot exclude the beneficial effects resulting from the ability of resveratrol to modulate UPS.

## 5. Conclusions

This study demonstrates that resveratrol attenuates the induction of cell senescence resulting from CuSO_4_ exposure. Such effects result from the ability of resveratrol to promote cellular adaptive mechanisms, such as autophagy upregulation, which sustain cellular proteostasis and confer cellular resistance to stress. Cellular proteostasis maintenance was found to be crucial to prevent the development of senescent phenotypes. These data also uncover molecular targets, the modulation of which likely prevents age-associated cell and tissue functional deterioration and improves human healthspan.

## Figures and Tables

**Figure 1:**
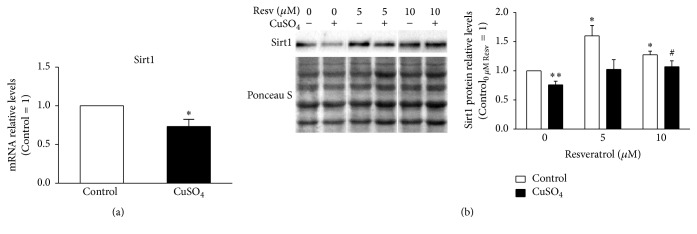
Reduced sirtuin 1 (Sirt1) expression in copper sulfate stress-induced premature senescent (CuSO_4_-SIPS) fibroblasts is restored by the addition of resveratrol. (a-b) WI-38 fibroblasts were incubated with 350 *µ*M CuSO_4_ (or Na_2_SO_4_, for controls) for 24 h. Then, the media were changed and cells were allowed to recover for an additional 72 h in the presence of 5 or 10 *µ*M resveratrol (or 0.1% dimethyl sulfoxide [DMSO] for controls). After this recovery period, fibroblasts were processed for different assays. (a) Sirt1 transcript levels were assessed by quantitative (q)PCR and plotted with the assumption that control mRNA levels equaled 1. TATA box binding protein (TBP) was the selected housekeeping gene. (b) Sirt1 relative protein content was determined by western blot, using Ponceau S staining to normalize protein loading. Depicted blots are representative and densitometric quantification is plotted with the assumption that control cells in the absence of resveratrol represent a relative protein level of 1. Data are presented as means ± SEM of at least three independent experiments. ^*∗*^*p* < 0.05 and ^*∗∗*^*p* < 0.01, when compared to control cells in the absence of resveratrol; ^#^*p* < 0.05, relative to CuSO_4_-treated cells without resveratrol.

**Figure 2:**
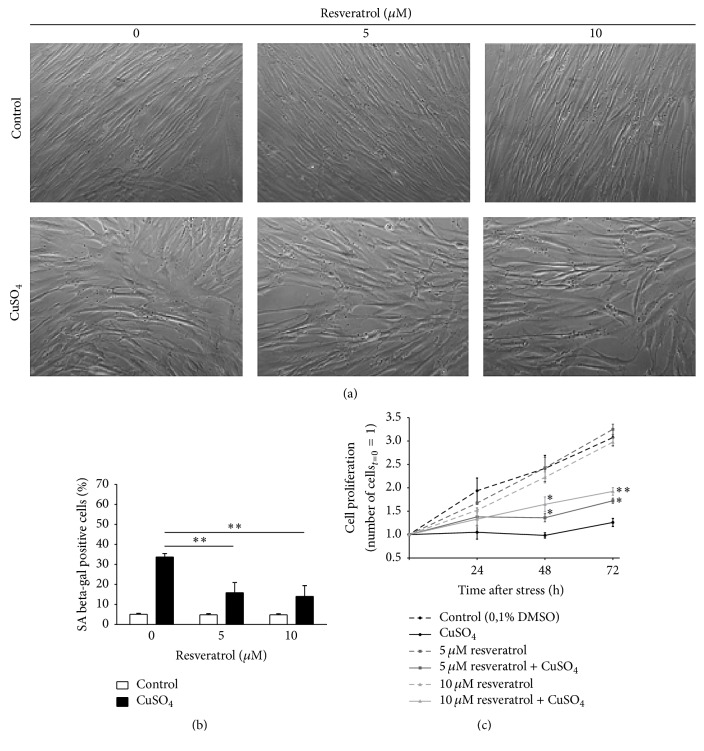
Resveratrol attenuates the appearance of typical senescence-associated features induced by CuSO_4_. (a) Cell morphology was evaluated 72 h after the removal of 350 *µ*M CuSO_4_ (or Na_2_SO_4_, for controls) in fibroblasts that were allowed to recover in the presence or absence of 5 or 10 *µ*M resveratrol. Representative images from the indicated conditions are depicted. (b) Senescence-associated beta-galactosidase (SA beta-gal) activity was detected 72 h after CuSO_4_ removal, and the percentage of positive cells was calculated for each condition after counting a minimum of 400 cells/well. (c) Cell proliferation was assessed by counting the viable cells in a Neubauer chamber at different time points after CuSO_4_ treatment (0, 24, 48, and 72 h). To facilitate direct comparisons between the indicated conditions over time, the number of viable cells on day 0 was assumed to be 1 for all treatments. Data are presented as means ± SEM of at least three independent experiments. ^*∗*^*p* < 0.05 and ^*∗∗*^*p* < 0.01, when compared to CuSO_4_-treated cells without resveratrol at the respective time point.

**Figure 3:**
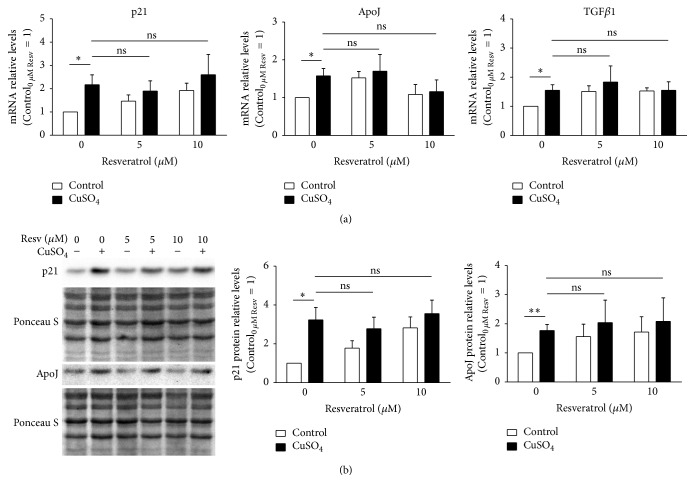
Resveratrol supplementation does not affect copper-induced expression of senescence-associated molecules. (a) Transcript-relative levels of cyclin-dependent kinase inhibitor 1A (p21), apolipoprotein J (ApoJ), and transforming growth factor beta 1 (TGF*β*1) were assessed by qPCR in 350 *μ*M CuSO_4_-treated fibroblasts that were allowed to recover in the presence of the indicated doses of resveratrol. (b) Representative western blots of p21 and ApoJ protein levels are depicted; the resulting densitometric analysis, normalized for control cells in the absence of resveratrol, is plotted for each analyzed protein. Ponceau S staining was used to control for protein loading. Data are presented as means ± SEM of at least three independent experiments. ^*∗*^*p* < 0.05; ^*∗∗*^*p* < 0.01; and ^ns^nonsignificant, for the comparisons between the indicated groups.

**Figure 4:**
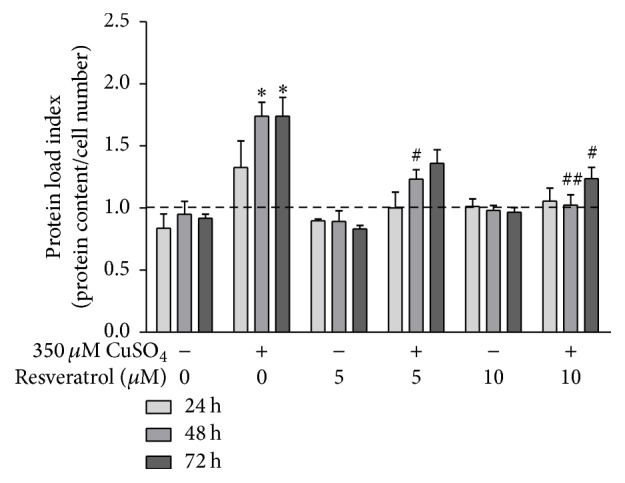
Imbalance in CuSO_4_-induced proteostasis is attenuated by resveratrol. The protein load index (PLI), used as a measure of cellular protein accumulation, was calculated as the ratio between total protein content and cell number for each condition at different time points after CuSO_4_ treatment (0, 24, 48, and 72 h). PLI values were normalized to the initial time point (0 h), and the relative values are plotted for the indicated conditions. Data are presented as means ± SEM of at least three independent experiments. ^*∗*^*p* < 0.05, when compared to control cells in the absence of resveratrol; ^#^*p* < 0.05 and ^##^*p* < 0.01, relative to CuSO_4_-treated cells without resveratrol, at the respective time points.

**Figure 5:**
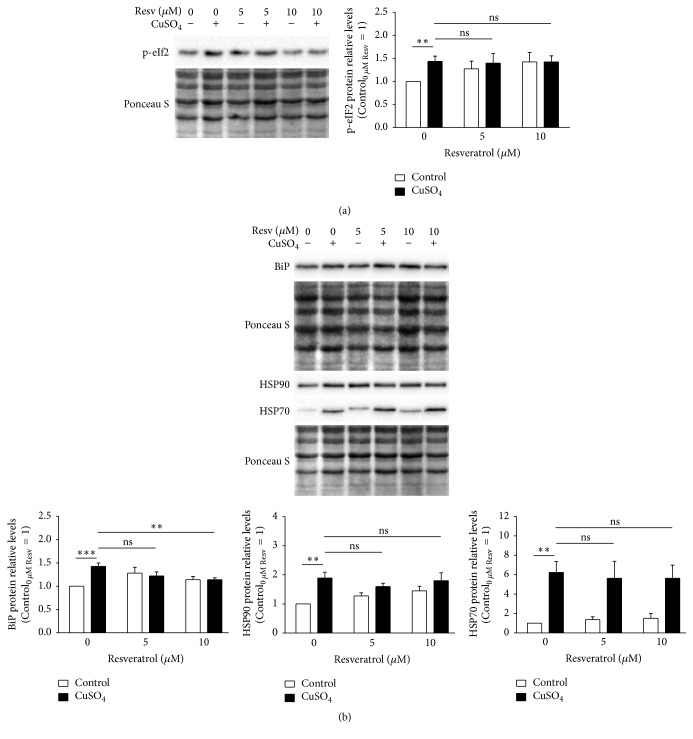
Resveratrol attenuates copper-induced immunoglobulin-binding protein (BiP) upregulation but has no effect on eukaryotic translation initiation factor 2 (eIF2) phosphorylation or heat shock protein 90 (HSP90) and HSP70 expression. (a) Phosphorylated eIF2 (p-eIF2) and (b) BiP, HSP90, and HSP70 relative protein levels were determined by western blot 72 h after the removal of 350 *μ*M CuSO_4_ (or Na_2_SO_4_, for controls) in fibroblasts that were allowed to recover in the presence or absence of resveratrol (5 or 10 *μ*M). Representative blots are depicted and densitometric quantification is plotted with the assumption that the protein level of each analyzed protein in control cells without resveratrol equaled 1. Ponceau S staining was used to normalize protein loading. Data are presented as means ± SEM of at least three independent experiments. ^*∗∗*^*p* < 0.01; ^*∗∗∗*^*p* < 0.001; and ^ns^nonsignificant, for the comparisons between the indicated groups.

**Figure 6:**
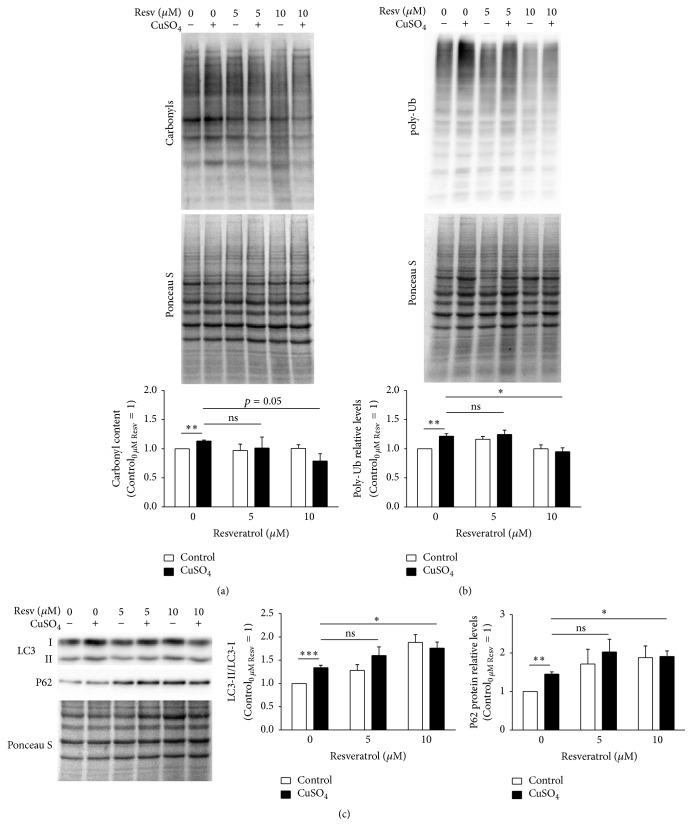
CuSO_4_-induced accumulation of carbonylated and polyubiquitinated proteins is reduced by resveratrol via induction of lysosomal autophagy. (a) Protein carbonyl content and (b) polyubiquitinated (poly-Ub) proteins were evaluated in fibroblasts exposed to the indicated conditions by western blot. Representative blots are depicted and densitometric quantification was normalized by assigning a value of 1 to the control cells in the absence of resveratrol. Ponceau S staining was used to account for differences in protein loading. (c) Lysosomal autophagy was studied by measuring the conversion of LC3-I to LC3-II, a critical step for autophagosome formation, and quantification of P62, a ubiquitin-binding protein that targets Ub substrates to autophagosomes. The LC3-II/LC3-I ratio and relative levels of P62 were evaluated via densitometric quantification and plotted with the assumption that control cells without resveratrol represent a value of 1. Data are presented as means ± SEM of at least three independent experiments. ^*∗*^*p* < 0.05; ^*∗∗*^*p* < 0.01; ^*∗∗∗*^*p* < 0.001; and ^ns^nonsignificant, for the comparisons between the indicated groups.

## References

[1] Matos L., Gouveia A. M., Almeida H. (2017). Resveratrol attenuates copper-induced senescence by improving cellular proteostasis. *Oxidative Medicine and Cellular Longevity*.

